# Thrombospondin-1 Partly Mediates the Cartilage Protective Effect of Adipose-Derived Mesenchymal Stem Cells in Osteoarthritis

**DOI:** 10.3389/fimmu.2017.01638

**Published:** 2017-11-29

**Authors:** Marie Maumus, Cristina Manferdini, Karine Toupet, Paul Chuchana, Louis Casteilla, Mélanie Gachet, Christian Jorgensen, Gina Lisignoli, Danièle Noël

**Affiliations:** ^1^INSERM, U1183, Hôpital Saint-Eloi, Montpellier, France; ^2^Montpellier University, UFR de Médecine, Montpellier, France; ^3^SC Laboratorio di Immunoreumatologia e Rigenerazione Tissutale, Istituto Ortopedico Rizzoli, Bologna, Italy; ^4^UMR STROMALab, CNRS 5273, INSERM U1031, Université Toulouse III-Paul Sabatier, Toulouse, France; ^5^Clinical Immunology and Osteoarticular Diseases Therapeutic Unit, Hôpital Lapeyronie, Montpellier, France

**Keywords:** mesenchymal stem cells, trophic factors, thrombospondin, cell therapy, osteoarthritis, chondrogenesis

## Abstract

**Objective:**

Assuming that mesenchymal stem cells (MSCs) respond to the osteoarthritic joint environment to exert a chondroprotective effect, we aimed at investigating the molecular response setup by MSCs after priming by osteoarthritic chondrocytes in cocultures.

**Methods:**

We used primary human osteoarthritic chondrocytes and adipose stem cells (ASCs) in mono- and cocultures and performed a high-throughput secretome analysis. Among secreted proteins differentially induced in cocultures, we identified thrombospondin-1 (THBS1) as a potential candidate that could be involved in the chondroprotective effect of ASCs.

**Results:**

Secretome analysis revealed significant induction of THBS1 in ASCs/chondrocytes cocultures at mRNA and protein levels. We showed that THBS1 was upregulated at late stages of MSC differentiation toward chondrocytes and that recombinant THBS1 (rTHBS1) exerted a prochondrogenic effect on MSC indicating a role of THBS1 during chondrogenesis. However, compared to control ASCs, siTHBS1-transfected ASCs did not decrease the expression of hypertrophic and inflammatory markers in osteoarthritic chondrocytes, suggesting that THBS1 was not involved in the reversion of osteoarthritic phenotype. Nevertheless, downregulation of THBS1 in ASCs reduced their immunosuppressive activity, which was consistent with the anti-inflammatory role of rTHBS1 on T lymphocytes. THBS1 function was then evaluated in the collagenase-induced OA model by comparing siTHBS1-transfected and control ASCs. The protective effect of ASCs evaluated by histological and histomorphological analysis of cartilage and bone was not seen with siTHBS1-transfected ASCs.

**Conclusion:**

Our data suggest that THBS1 did not exert a direct protective effect on chondrocytes but might reduce inflammation, subsequently explaining the therapeutic effect of ASCs in OA.

## Introduction

Osteoarthritis (OA) is the most prevalent rheumatic disease characterized by low chronic inflammation and major structural changes of the joint, causing pain, and functional disability. OA is more common in women than in men, and the prevalence of OA increases sharply with age but malalignment, obesity, genetics, and traumas are other associated risk factors (1). Hip and knee OA contributes the most to OA burden. Radiographic evidence of knee OA is observed in approximately 30% of men and women over the age of 65. Worldwide estimates are that 9.6% of men and 18.0% of women over the age of 60 years have symptomatic OA along with limitations in movement, and reductions in major activities of daily life (World Health Organization, see http://www.who.int/chp/topics/rheumatic/en/). No effective disease-modifying drug of OA is available. The current management relies on symptomatic pain relief and ultimately joint replacement surgery (2). OA of the knees and hips has become a major public health problem in the last years and is the third most prevalent musculoskeletal disorder (3). Development of innovative strategies to reduce the burden of OA and improve the management of the disease is therefore critical (4).

Mesenchymal stem cell (MSC)-based therapy has become an attractive strategy for OA treatment. MSCs are mesenchymal progenitor cells that can be isolated from various tissues such as bone marrow, adipose tissue, umbilical cord, and synovial tissue. They are characterized by their phenotypic profile, tripotent differentiation potential and trophic functions (5). Thanks to these properties, the therapeutic effectiveness of MSCs is extensively investigated in a wide range of diseases, including rheumatic disorders. In OA, single or repeated injections of MSCs from bone marrow (BM-MSCs), adipose [adipose stem cells (ASCs)], or synovial tissues were shown to efficiently protect mice, rabbits, or rats from cartilage degradation (6–9). In the clinics, several phase I studies have reported safety of intra-articular injection of autologous BM-MSCs or ASCs in knees of OA patients and encouraging results on pain and function (10–13). Among the proposed mechanisms, anti-inflammatory and chondroprotective effects mediated by prostaglandin E2 and hepatocyte growth factor have been proposed (14, 15). However, no demonstration has been shown *in vivo*.

It is well known that the immunosuppressive function of MSCs is induced by an inflammatory environment and the hypothesis that MSCs adapt to their environment to propose the appropriate response is now accepted (16). We recently showed that ASCs display chondroprotective and anti-inflammatory function both *in vitro* and *in vivo* (14–16). We therefore hypothesized that ASCs respond to the OA joint environment to exert a chondroprotective effect (8, 17) and investigated the molecular response setup by ASCs. We used primary human OA chondrocytes and ASCs in mono- and cocultures and performed a high-throughput secretome comparative analysis. Among differentially secreted proteins induced in coculture, we identified thrombospondin-1 (THBS1) as a potential candidate involved in the chondroprotective effect of ASCs and explored its function both *in vitro* and *in vivo* in the collagenase-induced osteoarthritis (CIOA) model (18).

## Materials and Methods

### Cell Culture

Primary cells (chondrocytes, synoviocytes, ASCs, and MSCs) were isolated from healthy or OA patients after informed consent. All subjects gave written consent in accordance with the Declaration of Helsinki. This study was carried out in accordance with the recommendations of Committee for Person Protection of Languedoc-Roussillon and approved by the French Ministry of Higher Education and Research (registration number: DC-2009-1052 and DC-2008-417). Protocols for isolation and characterization are described elsewhere (15). Typically, ASCs and MSCs were characterized by the expression of classical markers: CD13, CD73, CD90, and CD105 and the absence of hematopoietic and endothelial markers: HLA-DR, CD11b, CD14, CD31, CD34, CD45, and CD106. They were also shown to differentiate into the three lineages: adipocytes, osteoblasts, and chondrocytes [as previously shown in Ref. (19)].

### Coculture Assay

Chondrocytes or synoviocytes were plated at high density (500,000 cells/well) on the bottom of 6-well plates and cultured with ASCs (70,000 cells/insert) (ratio 7:1) in cell culture inserts (PET membranes, 0.4 µm pore porosity, BD Biosciences, Le Pont de Claix). Cultures were maintained for 7 days in 3 mL of minimal medium [DMEM supplemented with penicillin (100 U/mL), streptomycin (100 µg/mL), proline (0.35 mmol/L), ascorbic acid (0.17 mmol/L), and sodium pyruvate (1 mmol/L)] as previously described (15). In some experiments, chondrocytes were treated with human recombinant THBS1 (rTHBS1) (R&D Systems, Lille) at different concentrations in minimal medium during 7 days. Chondrocytes and ASCs were collected for RT-qPCR analysis and supernatants were stored at −20°C.

### Secretome Analysis

Chondrocyte and ASC monocultures (10^6^ cells/60 mm culture dish) or cocultures (5 × 10^5^ each cell type/60 mm culture dish) were maintained in αMEM containing 2% platelet lysate overnight. Three different cell samples were used. They were then washed five times with PBS to eliminate platelet lysate (Macopharma, Tourcoing) and 2.5 mL of minimal medium were added. After 48 h, supernatants from cocultures and chondrocyte or ASC monocultures were centrifuged (300 *g*, 5 min), filtered (0.22 µm), and stored at −80°C until secretome analysis. Secretome analysis was performed on coculture supernatants and on mixed monoculture supernatants. The rationale for mixing monoculture supernatants was to accurately compare levels of proteins secreted by each cell type in non-induced conditions to those secreted in cocultures upon crosstalk. Proteins were concentrated by TCA-NLS precipitation and quantified by Lowry method (Bio-Rad DC) as described previously (20). Reduced/alkylated protein samples were separated on gel Nupage 4–12% and stained by Coomassie blue (Instant Blue, Invitrogen), cut into bands, and digested with trypsin. After extraction, peptides were analyzed by mass spectrometry on NanoLC/ESI LTQ-Orbitrap Velos MS/MS. Results were analyzed by consulting protein data bases with Mascot Daemon software and proteins were validated with Prosper software. Spectral counting approach (score) was used to compare cocultures and mixed monocultures conditions. The mass spectrometry proteomics data have been deposited to the ProteomeXchange Consortium *via* the PRIDE [1] partner repository with the dataset identifier PXD008146.

### Chondrogenic Differentiation

Chondrogenic differentiation was induced by centrifuging 2.5 × 10^5^ MSCs at 300 *g* for 5 min in 15 mL conical tubes. Chondrogenic medium (DMEM high glucose, dexamethasone 0.1 µM, sodium pyruvate 1 mM, ascorbic-2-phosphate acid 170 µM, proline 0.35 mM, ITS, TGF-β3 at 10 ng/mL) was changed every 3 days for 21 days. When tested, human rTHBS1 (10 or 100 ng/mL) was added in the medium at day 14 for the 7 last days of differentiation. At day 21, micropellets were washed in PBS and immediately processed or stored at −80°C.

### Cell Transfection Protocol

Adipose stem cells were transfected at 60% of confluence with 100 nM of siRNA control (si*CT*) or *THBS1* (si*THBS1*) (Ambion, ThermoFisher Scientific, Illkirsch; see references in Table 1) using Oligofectamine reagent following the manufacturer’s instructions (Life Technologies, Courtaboeuf). Transfection was done on the day before coculture.

**Table 1:** List of primers and assays for PCR analysis and transfection experiments.

Primer sequences		

Gene	Sequence forward	Sequence reverse
*ACAN*	TCGAGGACAGCGAGGCC	TCGAGGGTGTAGCGTGTAGAGA
*ALPL*	CCACGTCTTCACATTTGGTG	GCAGTGAAGGGCTTCTTGTC
*COL10A1*	TGCTGCCACAAATACCCTTT	GTGGACCAGGAGTACCTTGC
*COL1A1*	CCTGGATGCCATCAAAGTCT	CGCCATACTCGAACTGGAAT
*COL2A1* variant B	CAGACGCTGGTGCTGCT	TCCTGGTTGCCGGACAT
*COL3A1*	CGCCCTCCTAATGGTCAAGG	AGGGCCTGAAGGACCAGCTT
*COX2*	CGGTGAAACTCTGGCTAGACAG	GCAAACCGTAGATGCTCAGGGA
*HAPLN1*	TTCCACAAGCACAAACTTTACACAT	GTGAAACTGAGTTTTGTATAACCTCTCAGT
*IDO1*	GCCTGATCTCATAGAGTCTGGC	TGCATCCCAGAACTAGACGTGC
*IL6*	AGACAGCCACTCACCTCTTCAG	TTCTGCCAGTGCCTCTTTGCTG
*IL8*	GAGAGTGATTGAGAGTGGACCAC	CACAACCCTCTGCACCCAGTTT
*MMP13*	GACTTCCCAGGAATTGGTGA	TACCCCAAATGCTCTTCAGG
*RSP9*	ATGAAGGACGGGATGTTCAC	GATTACATCCTGGGCCTGAA
*SOX9*	AGGTGCTCAAAGGCTACGAC	GTAATCCGGGTGGTCCTTCT
*THBS5*	CCGACAGCAACGTGGTCTT	CAGGTTGGCCCAGATGATG
*TNFα*	AGCCCATGTTGTAGCAAACCCTC	TGGTTATCTCTCAGCTCCACGCCA
*TSG6*	TCACCTACGCAGAAGCTAAGGC	TCCAACTCTGCCCTTAGCCATC

**TaqMan^®^ Gene Expression Assay ID**		

**Gene**	**ID**	

*ARG1*	Hs00163660_m1	
*COL4A1*	Hs01098873_m1	
*CSTA*	Hs00193257_m1	
*EDIL3*	Hs00174781_m1	
*IGFBP5*	Hs00181213_m1	
*NDRG1*	Hs00608387_m1	
*PTX3*	Hs00173615_m1	
*PYCARD*	Hs01547324_m1	
*RPS9*	Hs02339424_m1	
*SRGN*	Hs01004159_m1	
*THBS1*	Hs00962908_m1	
*THBS2*	Hs01568063_m1	
*THBS3*	Hs00938498_m1	
*THBS4*	Hs00170261_m1	
*UCHL3*	Hs00234683_m1	
*XYLT1*	Hs00544498_m1	

**siRNA references**		

**Target**	**ID**	

*siCT*	Ambion^®^, 4390844	
*siTHBS1*	Ambion^®^, s14098	

*THBS1, thrombospondin-1*.

### Splenocyte Proliferative Assay

Splenocytes were collected from spleens of adult C57Bl6 mice and suspended in IMDM glutamax medium supplemented with 10% inactivated fetal calf serum, 2 mM glutamine, 100 U/mL penicillin, and 100 µg/mL streptomycin, 0.1 mM nonessential amino acids, 1 mM sodium pyruvate, 20 mM HEPES (*N*-2-hydroxyethylpiperazine-*N*′-2-ethanesulfonic acid), and 5 × 10^5^ M 2-mercaptoethanol. Human ASCs transfected with siCT or siTHBS1 were plated in 96-well flat-bottom plates at different density (5 × 10^3^, 1 × 10^4^, or 2 × 10^4^ cells/well). Splenocytes were added at 2 × 10^5^ cells/100 μL/well and mitogen-driven proliferation of T lymphocytes was induced by adding 5 µg/mL concanavalin A (ConA; Sigma-Aldrich, Saint-Quentin-Fallavier). When tested, rTHBS1 was added at different concentrations with stimulated splenocytes. Unstimulated splenocytes were used as negative control. After 3 days incubation, splenocyte proliferation was quantified using CellTiter-Glo Luminescent Cell Viability Assay (Promega, Charbonnières-les-Bains) following the manufacturer’s instructions. The inhibitory effect of ASCs on splenocyte proliferation was quantified subtracting the signal of unstimulated splenocytes and proliferation rate was calculated referring to 100% the value of ConA-stimulated splenocytes.

### RT-qPCR

Total RNA was extracted from cells using the RNeasy kit (Qiagen, Courtaboeuf). RNA (0.5 µg) was reverse transcribed using the M-MLV reverse transcriptase (ThermoFisher scientific). PCR reaction was performed as previously described (15). All details for primer sequences (SYBR Green or TaqMan technologies) are described in Table 1. All values were normalized to RPS9 housekeeping gene since expression of this gene was the most constant among three others evaluated (GAPDH, β2 microglobulin, and β actin). Values were expressed as relative expression or fold change using the respective formulae 2^−ΔCT^ or 2^−ΔΔCt^.

### Quantification of Secreted Factors

Supernatants from ASC/synoviocyte or ASC/chondrocyte cocultures were used to quantify the concentrations of interleukin (IL)-6, CXCL8/IL-8, CCL2/MCP-1, CCL3/MIP-1α, and CCL5/RANTES using multiplex bead-based sandwich immunoassay kits (Bio-Rad, Marnes-la-Coquette) following the manufacturer’s instructions. THBS1 was detected by specific Enzyme-linked ImmunoSorbent Assays (ELISA; R&D Systems).

### CIOA Mouse Model

The study was conducted in accordance with guidelines and regulations of the Ethical Committee for animal experimentation of the Languedoc-Roussillon (Approval 5349-2016050918198875). All experiments were performed after final approval given by the French Ministry for Education, Higher Education and Research. CIOA model was performed as previously described (17). Briefly, both knee joints of mice were injected with 1 U collagenase type VII from *Clostridium histolyticum* (Sigma-Aldrich) in 5 µL of saline at day 0 and day 2, causing disruption of the ligaments and local instability of the joint. At day 7, groups of 20 mice received saline, ASC-siCT, or ASC-siTHBS1 (2 × 10^5^ cells/5 μL of saline solution). Mice were sacrificed at day 42. Hind paws were collected and fixed in formaldehyde 4% for 7 days before histological processing.

### Histological Analysis

After fixation, left hind paws were decalcified (formic acid 5%, 2 weeks), embedded in paraffin, cut (three slices of 7 µm each 140 µm), and stained with safranin O fast green staining. Cartilage degradation was quantified using the modified Pritzker OARSI score as described (17).

### Microtomography Analysis

Right hind paws were dissected to carefully remove smooth tissues and expose articular cartilage of tibiae. Tibiae were scanned in a microCT scanner (SkyScan 1176, resolution 9 µm, 0.5 mm aluminum filter). Assessment of bone parameters was performed using NRecon and CTan softwares (SkyScan).

### Confocal Laser Scanning Microscopy

Quantification of cartilage damage was assessed after scanning the entire articular cartilage of tibiae through their depth in XYZ-mode, with a confocal laser scanning microscope (CLSM; TCS SP5-II, Leica Microsystems, Nanterre, France) with a voxel size of 6 µm, a 5× dry objective and a UV laser light source (l¼ 405 nm). Image stacks were then processed to evaluate articular cartilage degradations. Assessment of cartilage morphometric parameters was performed in both lateral and medial plateau of each tibia using Avizo software (FEI Visualization Sciences Group, Lyon).

### Statistical Analysis

Data were expressed as the mean ± SEM. Statistical analysis was performed using the GraphPad Prism software. The comparison between two different unpaired groups was analyzed with a Mann–Whitney test for a non-Gaussian distribution and with an unpaired *t*-test for a Gaussian distribution. The comparison between several groups was analyzed with a Kruskal–Wallis test or an ANOVA.

## Results

### THBS1 Selection from MSC Secretome Analysis and Quantification of THBS1 Secretion in Osteoarthritic Chondrocyte/ASC Cocultures

**Figure 1:**
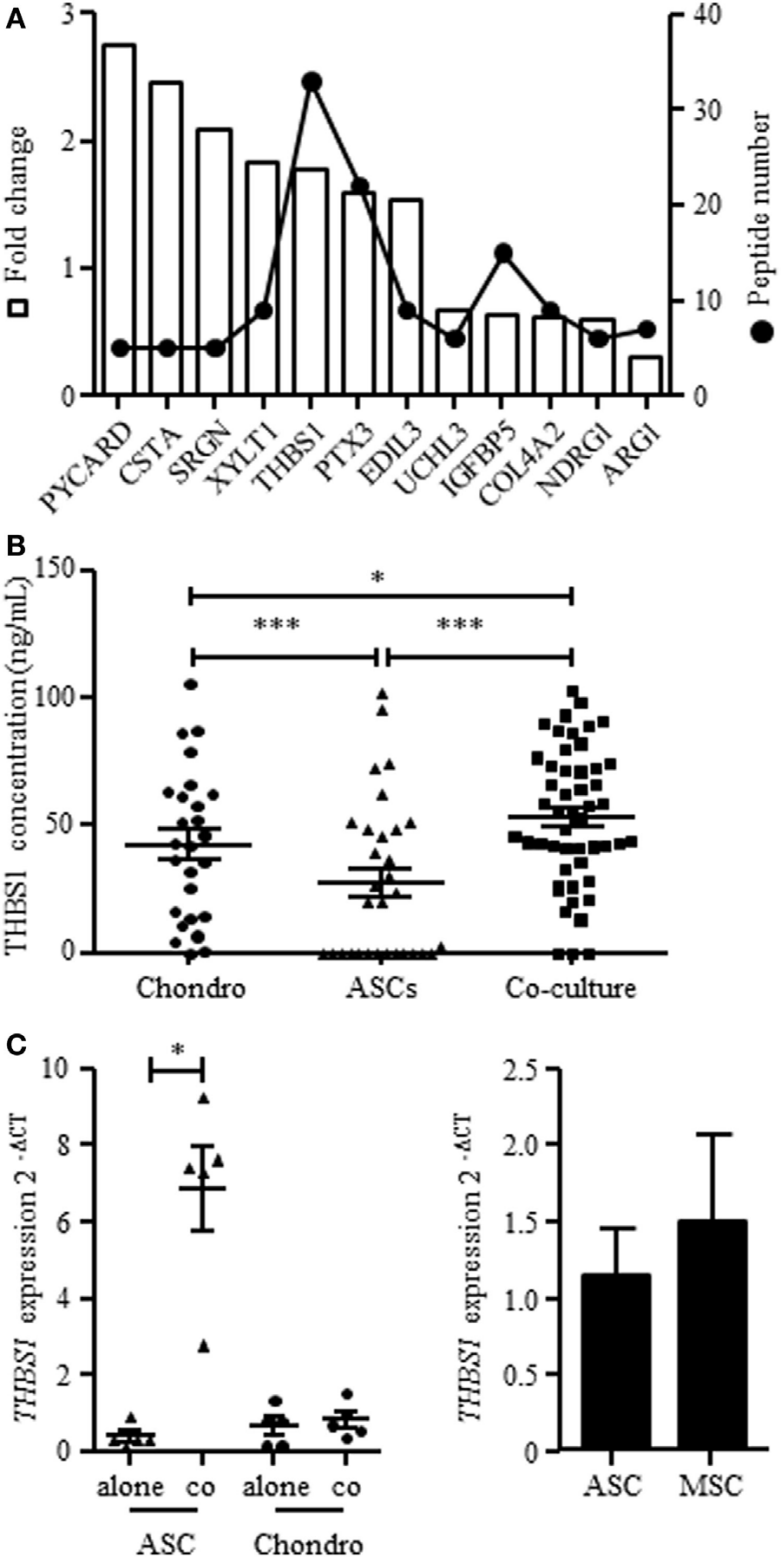
Expression of THBS1 in chondrocyte and adipose stem cell (ASC) mono- or cocultures. **(A)** Proteins identified in the secretome of chondrocyte/ASC cocultures significantly differentially secreted in chondrocyte/ASC cocultures as compared to mixed monocultures. The white bars represented the fold change of protein expression in coculture and the dot plot represented the number of quantified peptides for each proteins. Abbreviations: *PYCARD*, PYD and CARD domain containing; *CSTA*, cystatin A; *SRGN*, serglycin; *XYLT1*, xylosyltransferase 1; *THBS1*, thrombospondin-1; *PTX3*, pentraxin-3; *EDIL3*, EGF-like repeats and discoidin domains 3; *UCHL3*, ubiquitin C-terminal hydrolase L3; *IGFBP5*, insulin-like growth factor binding protein 5; *COL4A2*, collagen type IV alpha 2; *NDRG1*, N-Myc downstream regulated 1; *ARG1*, arginase 1. **(B)** THBS1 protein was quantified in the supernatants of chondrocyte monocultures (Chondro), ASC monocultures (ASC) or cocultures by Enzyme-linked ImmunoSorbent Assay. Results are expressed as the mean concentration ± SEM (*n* = 26–50 patient cell replicates). **(C)** *THBS1* mRNA level was measured in ASCs and in chondrocytes, cultured alone or in cocultures by RT-qPCR. Results are expressed as relative gene expression (2^−ΔCT^) and represented as mean ± SEM (*n* = 5 patient cell replicates). **(D)** *THBS1* mRNA level was measured in BM-MSCs and ASCs by RT-qPCR (*n* = 5). Statistics used unpaired *t*-test **(B)** or Mann–Whitney test **(C,D)**: *p ≤ 0.05, ***p ≤ 0.001.

We here aimed at identifying factors that could mediate the chondroprotective and anti-inflammatory functions of ASCs that we have previously described *in vitro* (14, 15) using secretome analysis. We compared proteins produced in the supernatants from chondrocyte/ASC coculture versus supernatants from either monoculture or mixed supernatants from both monocultures. Comparison between cocultures and mixed supernatants aimed at better selecting proteins that were modulated by the crosstalk between cells. We identified 2,043 proteins, among which 62% were predicted to be secreted. Ninety-two proteins were statistically different between cocultures and mixed monocultures. We selected only 12 proteins on the basis of their identification by more than 1 peptide and of a potentially relevant function (Figure 1A; Table 2). Validation of gene expression for these 12 proteins has been performed by RT-qPCR (Figure S1A in Supplementary Material) but only 3 were significantly induced in ASCs when cocultured with chondrocytes (Figure S1B in Supplementary Material). Finally, we selected THBS1 because it was the highest induced protein in cocultures, and identified by the greater number of peptides (Figure 1A). THBS1 was also chosen because its over-expression was previously reported to be protective in a rat model of OA and its absence induced skeletal abnormalities in knock-out mice (21, 22). We therefore hypothesized that the chondroprotective effect of ASCs in OA could be mediated by THBS1.

**Table 2:** Proteins identified in the secretome of chondrocyte/adipose stem cells (ASC) cocultures.

Gene name	Number of quantified peptides	Protein coverage (%)	Median score of coculture	Median score of mix	Ratio coculture/mix
*PYCARD*	5	16.4	361	131	2.76
*CSTA*	5	39.8	245	100	2.45
*SRGN*	5	10.1	230	110	2.09
*XYLT1*	9	3.4	238	130	1.83
*THBS1*	33	22.0	1,343	759	1.77
*PTX3*	22	41.5	1,341	841	1.59
*EDIL3*	9	13.8	356	233	1.53
*UCHL3*	6	24.4	128	192	0.67
*IGFBP5*	15	40.8	260	406	0.64
*COL4A2*	9	2.9	122	199	0.61
*NDRG1*	6	4.1	67	112	0.60
*ARG1*	7	5.6	43	139	0.31

*Secretome analysis compared proteins produced in the supernatants from chondrocyte/ASC coculture versus supernatants from either monoculture or mixed supernatants from both monocultures. Among 2043 identified proteins by MS/MS, 92 were significantly differentially secreted in chondrocyte/ASC cocultures as compared to mixed monocultures and only 12 were selected for validation*.

*PYCARD, PYD and CARD domain containing; CSTA, cystatin A; SRGN, serglycin; XYLT1, xylosyltransferase 1; THBS1, thrombospondin-1; PTX3, pentraxin-3; EDIL3, EGF-like repeats and discoidin domains 3; UCHL3, ubiquitin C-terminal hydrolase L3; IGFBP5, insulin-like growth factor binding protein 5; COL4A2, collagen type IV alpha 2; NDRG1, N-Myc downstream regulated 1; ARG1, arginase 1*.

We first validated the findings of secretome analysis by determining the protein level of THBS1 in chondrocyte/ASC cocultures by quantitative ELISA. We confirmed a significant increase of THBS1 in cocultures as compared to chondrocyte or ASC monocultures (Figure 1B). We then determined the expression level of *THBS1* mRNA in chondrocytes and ASCs cultured independently or in cocultures. Expression of *THBS1* mRNA was similar in ASC and chondrocyte monocultures as well as in chondrocytes when cocultured with ASCs (Figure 1C). In contrast, *THBS1* mRNA was highly upregulated in ASCs when they were cocultured with chondrocytes. These data demonstrated that upregulation of THBS1 protein in ASC/chondrocyte cocultures was directly correlated with the upregulation of *THBS1* mRNA in ASCs.

### THBS1 Enhances the Chondrogenic Differentiation of MSCs

**Figure 2:**
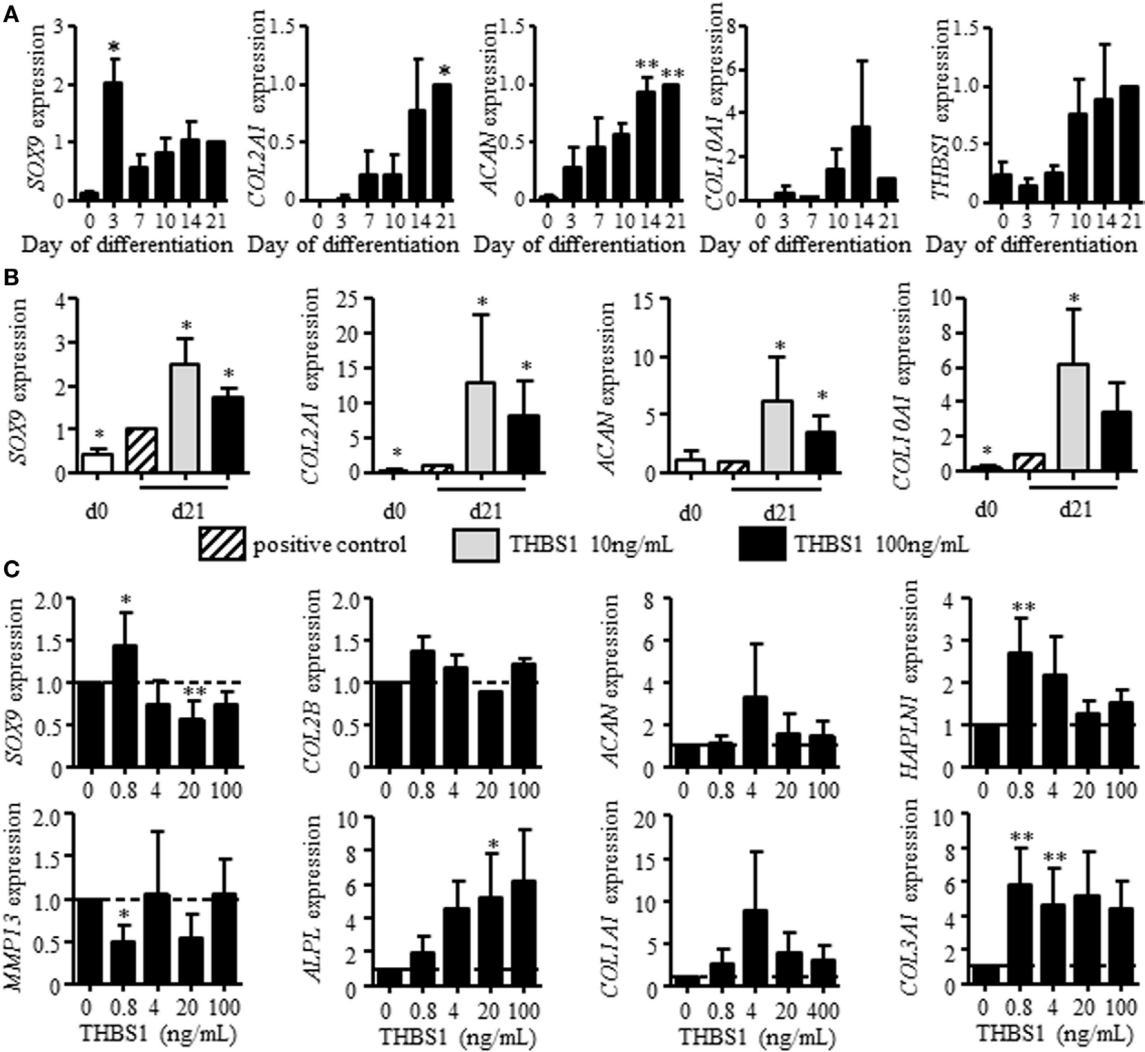
Effect of recombinant thrombospondin-1 (THBS1) on chondrogenesis and osteoarthritic chondrocyte phenotype. **(A)** Relative expression of markers specific for chondrocytes [*SOX9*, collagen type II variant B (*COL2A1*), aggrecan (*ACAN*), collagen type X (*COL10A1*)] or *THBS1* at different time points during differentiation of mesenchymal stem cells (MSCs) into chondrocytes or **(B)** after treatment with 10 or 100 ng/mL of THBS1 from days 14 to 21 of differentiation. Results are normalized to gene expression level in positive control (TGFβ3-induced differentiation of MSC) at day 21 and represented as mean ± SEM of four biological replicates. **(C)** Markers of mature chondrocytes (*SOX9, COL2A1 variant B, ACAN*, and *HAPLN1*), hypertrophic chondrocytes (*MMP13, ALPL*), and fibrocartilage (*COL1A1, COL3A1*) were quantified in osteoarthritic chondrocytes treated with different concentrations of recombinant THBS1 (rTHBS1) (*n* = 5 biological replicates). Results are expressed as fold change of gene expression compared to untreated chondrocytes and represented as mean ± SEM. Statistics used Mann–Whitney test: *p ≤ 0.05, **p ≤ 0.01.

We first evaluated whether THBS1 may be involved in chondrogenesis. Here, we used BM-MSCs instead of ASCs because of their higher potential to differentiate into chondrocytes and confirmed similar expression of THBS1 in the two cell types (Figure 1D). We investigated THBS1 expression in BM-MSCs induced to differentiate into chondrocytes by the standard micropellet technique and measured the expression of classical chondrocyte markers at different time points. All four markers *SOX9, COL2A1* variant B, *COL10A1*, and *ACAN* were upregulated after 21 days of pellet culture (Figure 2A). Although not significant, expression of *THBS1* was also upregulated by a fourfold factor at late time points. The direct effect of THBS1 was then tested by adding the recombinant protein at the end of the differentiation process (between days 14 and 21) when expression of endogenous THBS1 is increased. Two doses of rTHBS1 were evaluated. Compared to the positive control (BM-MSCs cultured in presence of TGFβ3), addition of either doses of rTHBS1 significantly upregulated the expression of all chondrocyte markers (*SOX9, COL2A1* variant B, *ACAN*, and *COL10A1*) by day 21 (Figure 2B). These results indicated that THBS1 enhanced BM-MSC differentiation into the chondrogenic pathway.

We then determined the potential effect of THBS1 on chondrocyte phenotype by evaluating the dose-dependent effect of rTHBS1 on OA primary chondrocytes and quantifying the expression of several chondrocyte markers by RT-qPCR. At low doses, rTHBS1 increased the expression of mature chondrocyte markers (*SOX9, COL2A1* variant B, *ACAN*, and *HAPLN1*), which was not observed for high doses of rTHBS1 (Figure 2C). *SOX9* expression was even significantly reduced at high doses of rTHBS1. Expression of the two hypertrophic chondrocyte markers *MMP13* and *ALPL* differed. *MMP13* was significantly decreased at the lowest dose of rTHBS1 but was not modulated at higher doses while *ALPL* increased in a dose-dependent manner. Finally, expression of two markers of fibrocartilage, *COL1A1* and *COL3A1*, was increased whatever was the concentration of rTHBS1 in the culture medium. Indeed, rTHBS1 did not positively impact the altered phenotype of osteoarthritic chondrocytes.

### THBS1 Secreted by ASCs Has No Protective Effect on Osteoarthritic Chondrocytes

**Figure 3:**
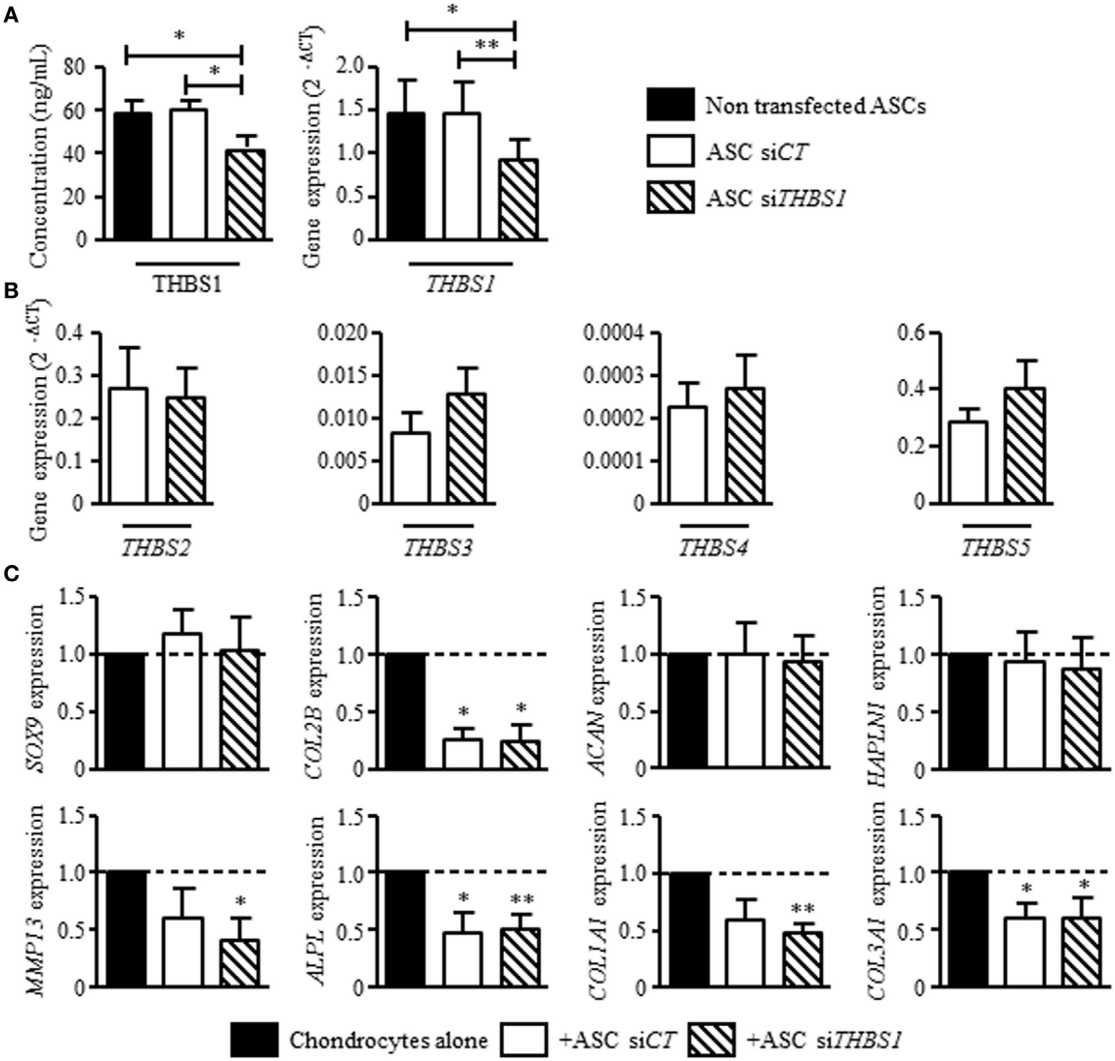
Effect of thrombospondin-1 (THBS1) secreted by adipose stem cells (ASCs) on osteoarthritic chondrocyte phenotype. **(A)** THBS1 protein concentration and mRNA expression in non-transfected ASCs, in ASCs transfected with si*CT* or si*THBS1*. **(B)** Expression of thrombospondin family members (*THBS2, THBS3, THBS4*, and *THBS5/COMP)* was quantified in ASC-si*CT* or ASC-si*THBS1*. Results are expressed as relative gene expression (2^−ΔCT^) and represented as mean ± SEM (*n* = 9 independent biological replicates). **(C)** Markers of mature chondrocytes (*SOX9, COL2A1 variant B, ACAN*, and *HAPLN1*), hypertrophic chondrocytes (*MMP13, ALPL*), and fibrocartilage (*COL1A1, COL3A1*) were quantified in osteoarthritic chondrocytes after coculture with ASC-si*CT* or ASC-si*THBS1* (*n* = 8 biological replicates). Results are expressed as fold change of gene expression compared to chondrocyte monocultures and represented as mean ± SEM. Statistics used Mann–Whitney test: *p ≤ 0.05, **p ≤ 0.01.

Since we previously reported that ASCs modulate the phenotype of osteoarthritic chondrocytes by decreasing the expression of hypertrophic and fibrotic markers (15), we investigated the effect of *THBS1* silencing in ASCs. Using a siRNA approach, we reproducibly downregulated *THBS1* expression in ASCs by 30–37%, at the mRNA and protein level, respectively, as compared to untransfected ASCs or ASCs transfected with a control siRNA (si*CT*) (Figure 3A). Since THBS1 is part of thrombospondin family which includes 5 members (THBS-1, -2, -3, -4, and -5/cartilage oligomeric matrix protein), we checked whether downregulation of THBS1 could induce a modulated expression of other thrombospondin members. Downregulation of *THBS1* expression in ASCs did not impact the expression of other thrombospondin family members (Figure 3B). As expected, addition of ASC-si*CT* decreased the expression of *MMP13, APLP, COL1A1*, and *COL3A1* in cocultured chondrocytes (Figure 3C). *COL2A1* variant B was also significantly reduced. By comparison, a similar effect of ASC-si*CT* and ASC-si*THBS1* was observed on chondrocytes indicating that THBS1 was not involved in the regulation of osteoarthritic chondrocyte metabolism by ASCs.

### THBS1 Does Not Impact the Secretory Profile of Osteoarthritic Synoviocytes or Chondrocytes but Exerts an Anti-inflammatory Effect on T Lymphocytes

**Figure 4:**
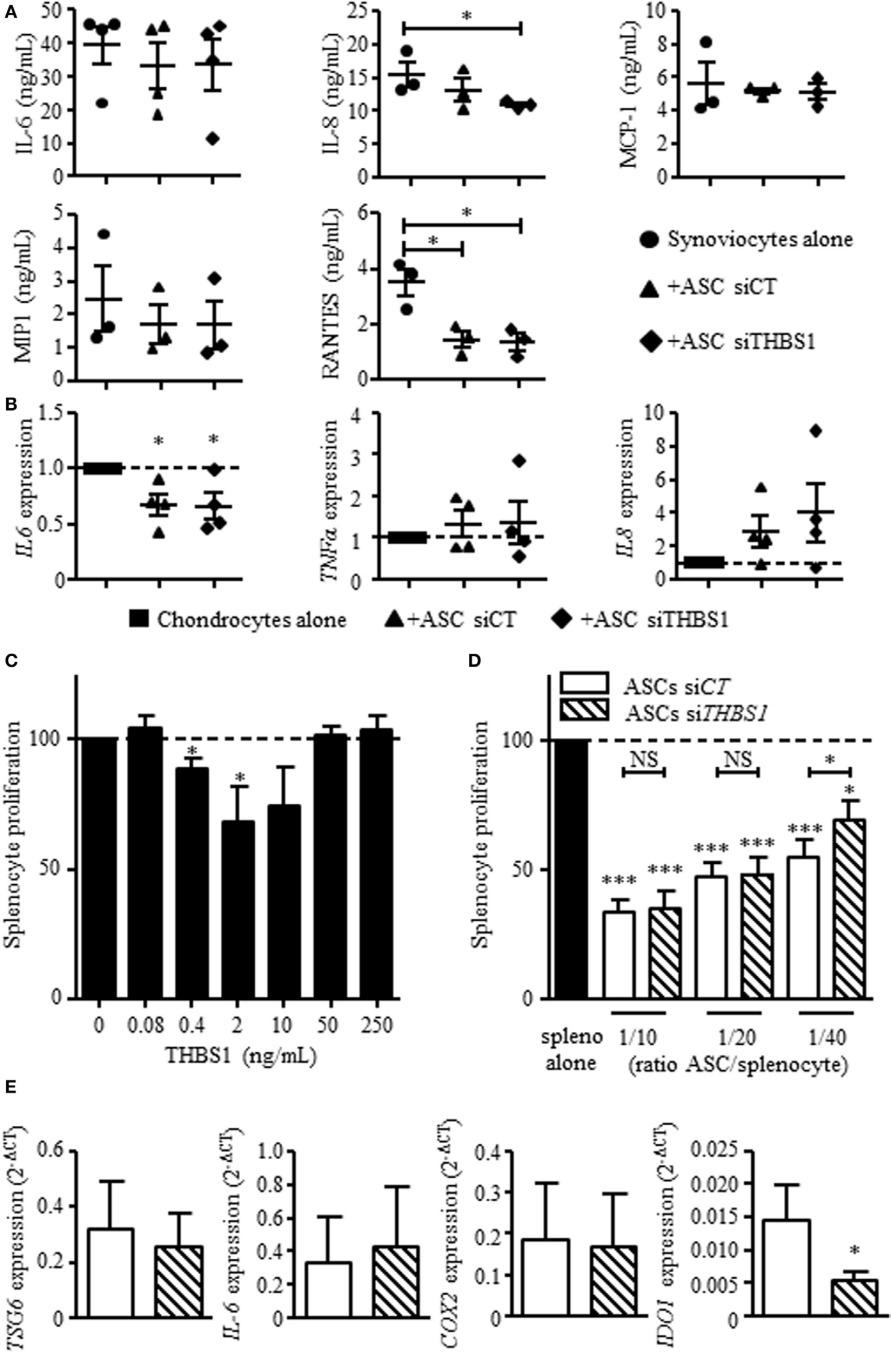
Effect of thrombospondin-1 (THBS1) on inflammatory cells. **(A)** Levels of pro-inflammatory proteins (IL-6, IL-8, MCP-1, MIP1, and RANTES) were quantified in the supernatants of synoviocytes cultured with or without adipose stem cell (ASC)-si*CT* or ASC-si*THBS1*. Results are expressed as the mean concentration of cytokine ± SEM (*n* = 3 biological replicates). **(B)** Pro-inflammatory cytokine expressions (*IL-6, TNFα*, and *IL-8*) was quantified in osteoarthritic chondrocytes after coculture with ASC-si*CT* or ASC-si*THBS1* (*n* = 4 biological replicates). Results are expressed as fold change of gene expression compared to untreated chondrocytes or chondrocyte monocultures and represented as mean ± SEM. **(C)** Role of THBS1 on T cell proliferation was quantified using murine splenocytes activated with ConA in presence of different concentrations of recombinant THBS1 (rTHBS1) or **(D)** activated murine splenocytes in presence of ASC-si*CT* or ASC-si*THBS1* at different ratios. Results are expressed as the percentage of ConA-induced proliferation of splenocytes which was assigned the value of 100% and represented as mean ± SEM (*n* = 6 independent biological replicates). **(E)** Immunosuppressive molecule expression (*TSG6, IL-6, COX2*, and *IDO1*) was quantified in ASC-si*CT* and ASC-si*THBS1*. Results are expressed as relative gene expression (2^−ΔCT^) and represented as mean ± SEM (*n* = 8 independent biological replicates). Statistics used Mann–Whitney test **(A,B,E)** or Kruskal–Wallis **(C,D)**: *p < 0.05, **p < 0.01, ***p < 0.001 compared to control.

Our previous results showed that ASCs reduce the secretion of inflammatory mediators by osteoarthritic synoviocytes and chondrocytes (14). We therefore determined whether THBS1 was involved in this effect using si*THBS1-*transfected ASCs. In coculture with synoviocytes, ASC-si*CT* tended to decrease the secretion of all tested factors but the decrease was only significant for RANTES (Figure 4A). Addition of ASC-si*THBS1* induced a similar reduction of inflammatory mediators. The level of IL-8 was even lower after addition of ASC-si*THBS1*. In coculture with chondrocytes, both ASC-si*CT* and ASC-si*THBS1* decreased expression of IL-6 and tended to increase expression of TNF-α and IL-8 in chondrocytes (Figure 4B). The results therefore showed that THBS1 was not involved in the modulation of the inflammatory profile of osteoarthritic synoviocytes or chondrocytes.

We then investigated the possible anti-inflammatory effect of THBS1 on T cell response. We added different doses of rTHBS1 in a T lymphocyte proliferative assay and measured the proliferation of ConA-activated T cells in the splenocyte population. A bell curve was obtained with no suppressive effect of rTHBS1 at the lowest and highest tested doses (Figure 4C). However, the doses of 0.4 and 2 ng/mL of rTHBS1 significantly reduced T lymphocyte proliferation. We then compared the suppressive capacity of ASC-si*CT* and ASC-si*THBS1* on T cell proliferation. Using different ASC/splenocyte ratios, we observed that ASC-si*THBS1* were significantly less suppressive than ASC-si*CT* at the lowest ratio (Figure 4D). We evaluated that the concentration of THBS1 was approximately 2.5 ng/mL at 1/40 ratio. This amount was in the range of the suppressive activity of rTHBS1 (see Figure 4C) while highest ratios exerted no effect, suggesting a dose-dependent effect of THBS1. In addition, silencing of THBS1 in ASCs did not affect the expression of the immunosuppressive molecules *TSG6, IL-6*, and *COX2* but significantly decreased *IDO1* expression (Figure 4E). Our results might therefore suggest that secretion of THBS1 at least partly mediated the anti-inflammatory effect of ASCs through the expression of *IDO1*.

### Lack of THBS1 Impairs the Chondroprotective Effect of ASCs *In Vivo*

**Figure 5:**
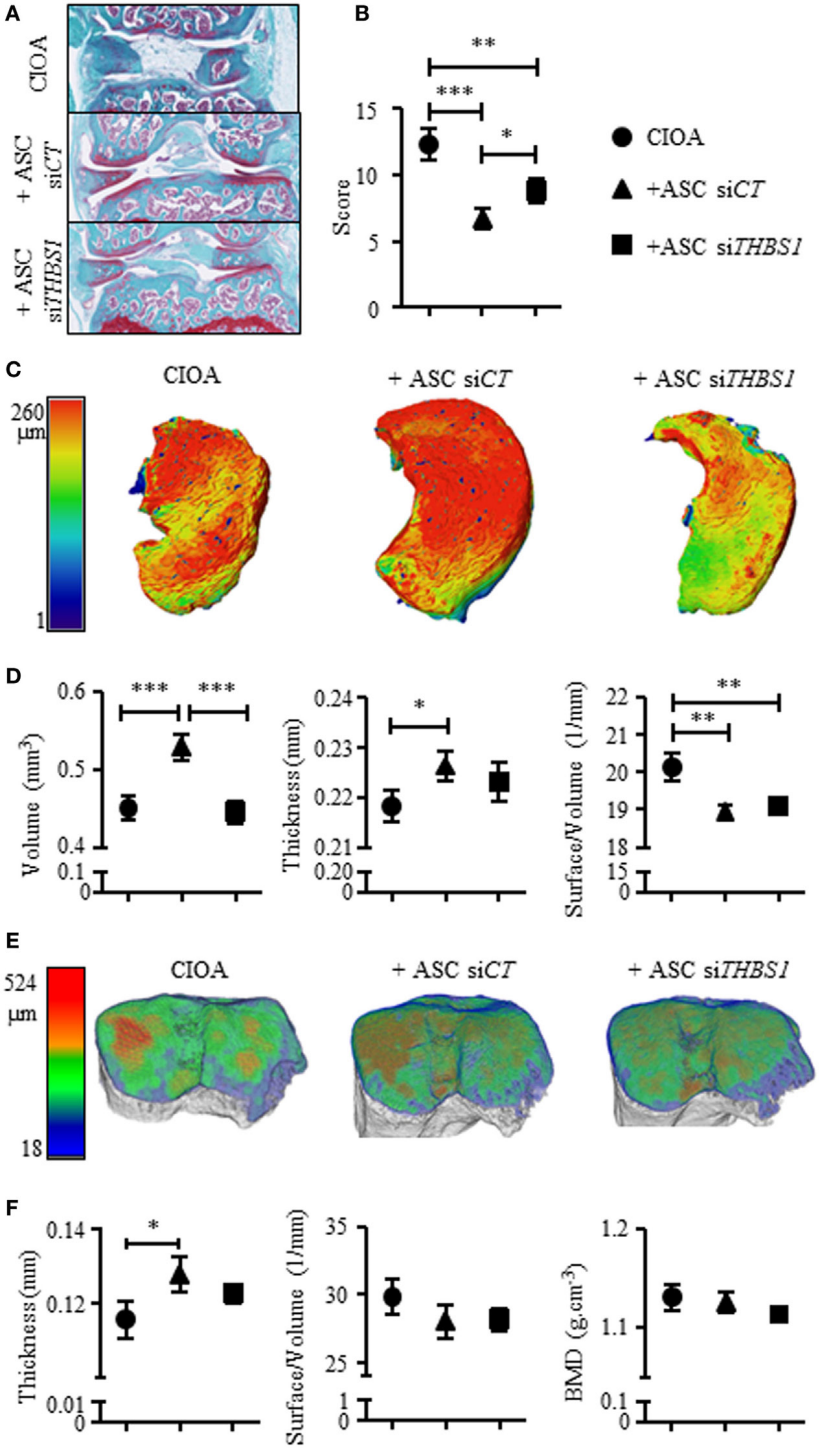
Effect of the downregulation of thrombospondin-1 (THBS1) in adipose stem cells (ASCs) in the collagenase-induced osteoarthritis (CIOA) murine model. **(A)** Representative photographs of knee joints from control CIOA mice (collagenase alone) (upper), and CIOA mice treated with ASC-si*CT* (middle) or ASC-si*THBS1* (lower) and **(B)** OA score for cartilage destruction as evaluated on histological sections of knee joints (*n* = 20/group). **(C)** Representative reconstructed 3D pictures of knee joint cartilage after confocal laser scanning microscopy analysis. A color bar code indicative of cartilage thickness was added on the left. **(D)** Cartilage morphometric parameters [cartilage volume, thickness, and surface irregularity (surface/volume)] were measured for the three groups of mice. **(E)** Representative reconstructed 3D pictures of tibiae epiphyses after microtomography analysis. A color bar code indicative of thickness was added on the left. **(F)** Bone morphometric analysis of sub-chondral bone measuring surface irregularity (surface/volume; 1/mm), thickness (mm), and bone mineral density (BMD; g/cm^3^) was performed. Results are expressed as mean ± SEM (*n* = 20/group). Statistics used ANOVA test: *p < 0.05, **p < 0.01, ***p < 0.001 compared to CIOA control group.

Finally, we determined whether THBS1 secretion plays a role in the anti-inflammatory and chondroprotective effect of ASCs in the CIOA pre-clinical model. To this end, we injected either ASC-si*CT* or ASC-si*THBS1* in the knee joints of mice induced to develop OA and evaluated cartilage and bone degradation at day 42 after OA induction. As previously shown (17), histological scoring on both femurs and tibias sections revealed that injection of human ASC-si*CT* protected mice from developing OA lesions as shown by representative pictures and OA score (Figures 5A,B). Histological analysis of knee joint sections revealed that ASC-si*THBS1* were significantly less efficient that ASC-si*CT* in protecting against cartilage degradation as shown by OA score (Figures 5A,B). Because histological analysis did not allow scoring the entire joint, we relied on morphometric analysis of cartilage using CLSM. Thanks to the autofluorescence property of cartilage, visualization and 3D reconstruction of entire articular cartilage on tibia plateaus are feasible. Using this technology, a highly significant preservation of cartilage volume was observed with ASC-si*CT* but not with ASC-si*THBS1* as shown in reconstructed 3D pictures (Figure 5C) and confirmed by volume quantification (Figure 5D). Other parameters, such as cartilage thickness and surface degradation (measured as surface/volume ratio), were preserved with both ASC-si*CT* and ASC-si*THBS1* even though ASC-si*THBS1* seemed to be less efficient (Figure 5D). At last, bone parameters were analyzed by μCT and 3D reconstructions of sub-chondral bone were performed using a color code for thickness representation. On 3D images of tibia epiphyses, sub-chondral bone thickness, as evaluated by larger red areas, was higher in mice treated with ASC-si*CT* compared to CIOA control or ASC-si*THBS1* treated mice (Figure 5E). These correlated with a significantly higher thickness measure of sub-chondral bone in ASC-si*CT* group compared to CIOA control (Figure 5F). Bone erosion (surface/volume) and bone mineral density parameters did not change in mice injected with ASC-si*CT* or ASC-si*THBS1* (Figure 5F). Altogether, the results suggested that ASCs expressing lower levels of THBS1 were less efficient to protect mice from developing OA symptoms.

## Discussion

In this study, we present evidence that *in vivo*, downregulation of THBS1 in ASCs impacted their chondroprotective effect in the CIOA model. This effect could be attributed to an anti-inflammatory role of THBS1 on the immune response and/or a chondroinductive effect of THBS1 on MSCs.

The main known function of the matricellular protein THBS1 is its anti-angiogenic and anti-inflammatory effect in various models, mainly in cancers and cardiac diseases (23). In experimental models of infarction, THBS1 was highly upregulated and THBS1^−/−^ mice exhibited inflammatory infiltrates into the infarcted heart (24). Mice lacking THBS1 also developed inflammation with a phenotype close to that observed in TGFβ null mice, had more severe course of acute colitis, and displayed colonic inflammation (25). This phenotype indicated a crucial anti-inflammatory function of THBS1 which might also be explained by its interaction with the NO pathway (26). However, THBS1 may exhibit anti- and pro-inflammatory effect depending on disease models and acts by a biphasic or dose-dependent mechanism (27). These contrasting roles are likely due to different interactions with various receptors or the presence of diverse proteins in the extracellular matrix of different tissues. In this study, we showed that rTHBS1 exerted an anti-proliferative role on T lymphocytes and that downregulation of THBS1 in ASCs decreased their immunosuppressive activity which could be related to lower expression of the immunosuppressive enzyme IDO1. Indeed, THBS1 might be a novel mediator of the immunosuppressive effect of ASCs and we proposed that THBS1 secreted by ASCs may exert a protective role in CIOA mice by decreasing the inflammatory immune response. This is supported by a previous work reporting adenoviral transfer of THBS1 (Ad-THBS1) in rats with anterior crucial ligament transection-mediated OA (21). Rats treated with Ad-THBS1 had less severe OA than controls and histological sections of knee joints revealed reduced microvessel density and inflammation. In another study using two rat models of OA, THBS1 was shown to induce regeneration of damaged cartilage (28). Similar results were described in other inflammatory models of arthritis such as the experimental model of erosive arthritis induced by injection of peptidoglycan-polysaccharide in rats. In this model, injection of a peptide-derived from THBS1 (residues D793-P824) prevented joint infiltration and inflammation and was associated with reduced serum levels of THBS1 (29). This effect was mediated by peptide interaction with neutrophils and the induction of CTGF in arthritis affected tissues (30). In rheumatoid arthritis patients, altered expression levels and tissue distribution of THBS1 was observed (31). Anti-tumor necrosis factor α (TNFα) therapy modulated THBS1 expression with inverse correlation with orphan nuclear receptor 4A2, IL8, and vascular endothelial growth factor expression levels suggesting that THBS1 may help restoring tissue homeostasis during inflammation resolution. These studies all argue for an anti-inflammatory role of THBS1, suggesting that THBS1 mediated at least in part the chondroprotective role of ASCs in the CIOA model through modulation of inflammation.

The other novel finding of our study was the role of THBS1 on chondrogenesis. When added at the late stage of MSC differentiation using the *in vitro* pellet culture model, THBS1 enhanced the production of markers for mature (SOX9, COL2A1 variant B, and ACAN) and hypertrophic (COL10A1) cartilage. Previous studies reported that THBS1 was highly expressed by chondrocytes and involved in the resistance of cartilage against endochondral ossification through its anti-angiogenic function (32). In miniature pigs, THBS1 treatment of cartilage lesions in femoral trochlea inhibited endochondral ossification, but failed to induce chondrogenesis. However, application of bone morphogenetic protein (BMP)-7 and THBS1 was shown to complement each factor in a functional manner. While BMP-7 induced chondrogenesis, THBS1 prevented hypertrophy and excessive endochondral ossification within the lesions (33). These data suggested that THBS1 upregulation during the hypertrophic stage of MSC differentiation might be related to its angiostatic function to prevent *in vivo* endochondral ossification. In this study, addition of THBS1 at late stages of differentiation enhanced chondrogenesis without inhibiting hypertrophy. However, interactions with other mediators likely occur *in vivo* to finely regulate THBS1 function. Our data are in line with another recent report on the role of THBS2 during chondrogenesis (34). The authors demonstrated that autocrine secretion of THBS2 promotes chondrogenesis *via* the Notch signaling pathway and attenuates hypertrophy. Since THBS1 and THBS2 are the most closely related members of THBS family, the finding that both members exhibited similar functions was indeed predicted. Impact of the prochondrogenic function of THBS1 secreted by ASCs in the CIOA model is, however, difficult to demonstrate. THBS1 might stimulate endogenous progenitors to produce cartilage and counteract tissue degradation occurring during OA. THBS1 might also enhance the differentiation of exogenously added MSCs and therefore stimulate cartilage regeneration/repair. This is, however, unlikely to occur since survival time of exogenous MSCs in immunocompetent mice is in the range of few days and MSCs are proposed to act principally through paracrine function rather than direct differentiation (17).

In conclusion, our data gather evidence that THBS1 exerts a prochondrogenic and anti-inflammatory function *in vitro*, which could explain the chondroprotective effect of ASCs in the CIOA model.

## Ethics Statement

Primary cells (chondrocytes, synoviocytes, ASCs, and MSCs) were isolated from healthy or OA patients after informed consent. All subjects gave written consent in accordance with the Declaration of Helsinki. This study was carried out in accordance with the recommendations of Committee for Person Protection of Languedoc-Roussillon and approved by the French Ministry of Higher Education and Research (registration number: DC-2009-1052 and DC-2008-417). The animal experimentation was conducted in accordance with guidelines and regulations of the Ethical Committee for animal experimentation of the Languedoc-Roussillon (Approval 5349-2016050918198875). All experiments were performed after final approval given by the French Ministry for Education, Higher Education and Research.

## Author Contributions

MM participated in the design of the study, acquisition, analysis and interpretation of data, manuscript preparation and final approval; CM, KT, PC, MG participated in acquisition and analysis of data, manuscript preparation and final approval. LC, CJ, GL participated in the design of the study, interpretation of data, manuscript preparation and final approval. DN carried out the conception and design of the study, participated in analysis and interpretation of data, wrote the manuscript.

## Conflict of Interest Statement

The authors declare that the research was conducted in the absence of any commercial or financial relationships that could be construed as a potential conflict of interest.

## Supplementary Material

Figure S1 Validation of secretome analysis. **(A)** Gene expression of proteins identified in the secretome of chondrocyte/ASC cocultures. Data represent the relative gene expression (2^−ΔCT^) in ASC monoculture (white bars), in chondrocyte monoculture (black bar), and in chondrocyte/ASC cocultures (gray bar). Results are represented as mean ± SEM (*n* = 3 biological replicates). Abbreviations: *PYCARD*, PYD and CARD domain containing; *CSTA*, cystatin A; *SRGN*, serglycin; *XYLT1*, xylosyltransferase 1; *THBS1*, thrombospondin-1; *PTX3*, pentraxin-3; *EDIL3*, EGF-like repeats and discoidin domains 3; *UCHL3*, ubiquitin C-terminal hydrolase L3; *IGFBP5*, insulin-like growth factor binding protein 5; *COL4A2*, collagen type IV alpha 2; *NDRG1*, N-Myc downstream regulated 1; *ARG1*, arginase 1. **(B)** SRGN, THBS1, PTX3, UCHL3, and NDRG1 mRNA level was measured in chondrocytes and in ASCs cultured alone or in cocultures (co) by RT-qPCR. Results are expressed as relative gene expression (2^−ΔCT^) and represented as mean ± SEM (*n* = 5 biological replicates). Statistics used Mann–Whitney test: **p* ≤ 0.05.
