# Supplementary figures and images for: Thrombospondin-1 Partly Mediates the Cartilage Protective Effect of Adipose-Derived Mesenchymal Stem Cells in Osteoarthritis
Source: Front Immunol. 2017 Nov 29;8:1638. doi: 10.3389/fimmu.2017.01638 (PMC5712679; doi:10.3389/fimmu.2017.01638)

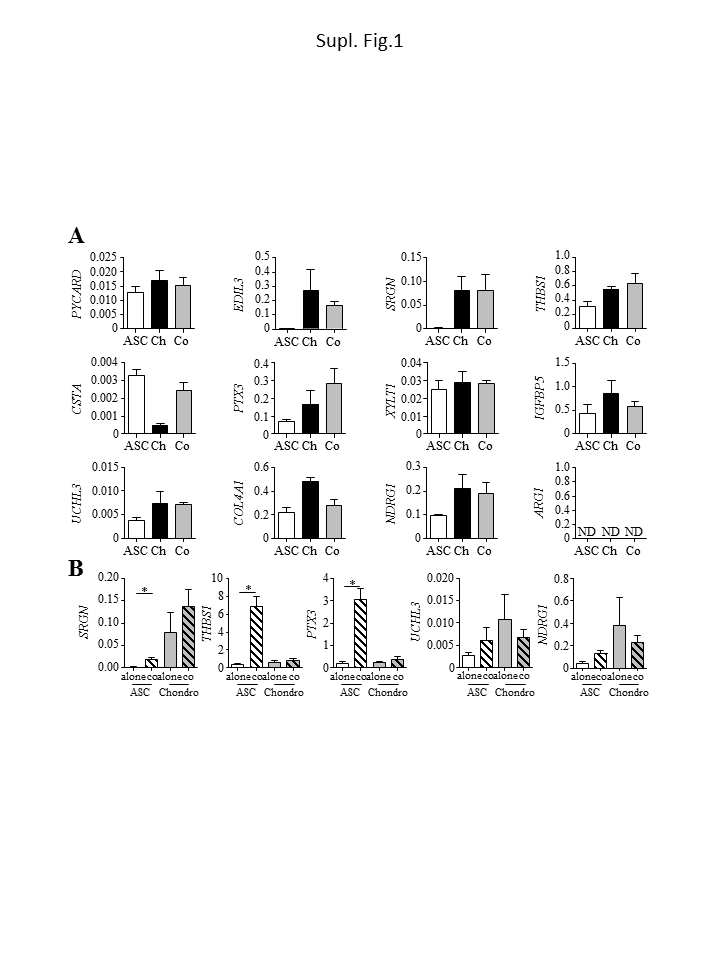

Supplement: Figure S1 — Validation of secretome analysis. (A) Gene expression of proteins identified in the secretome of chondrocyte/ASC cocultures. Data represent the relative gene expression (2−ΔCT) in ASC monoculture (white bars), in chondrocyte monoculture (black bar), and in chondrocyte/ASC cocultures (gray bar). Results are represented as mean ± SEM (n = 3 biological replicates). Abbreviations: PYCARD, PYD and CARD domain containing; CSTA, cystatin A; SRGN, serglycin; XYLT1, xylosyltransferase 1; THBS1, thrombospondin-1; PTX3, pentraxin-3; EDIL3, EGF-like repeats and discoidin domains 3; UCHL3, ubiquitin C-terminal hydrolase L3; IGFBP5, insulin-like growth factor binding protein 5; COL4A2, collagen type IV alpha 2; NDRG1, N-Myc downstream regulated 1; ARG1, arginase 1. (B) SRGN, THBS1, PTX3, UCHL3, and NDRG1 mRNA level was measured in chondrocytes and in ASCs cultured alone or in cocultures (co) by RT-qPCR. Results are expressed as relative gene expression (2−ΔCT) and represented as mean ± SEM (n = 5 biological replicates). Statistics used Mann–Whitney test: *p ≤ 0.05. [file image_1.tif]
